# *GATA3* Deletion Associated With Juvenile Idiopathic Arthritis: Expanding the Phenotypic Spectrum of Hypoparathyroidism, Sensorineural Deafness, and Renal Dysplasia (HDR) Syndrome

**DOI:** 10.1002/ajmg.a.64299

**Published:** 2025-11-05

**Authors:** Lauren N. Meiss, Amanda V. Karam, Julia Shalen, David E. Tunkel, Belina Y. Yi, Kelsey S. Guthrie, Emily M. Kudalkar, Anna E. Patrick, Sonja A. Rasmussen

**Affiliations:** 1 Department of Genetic Medicine, Johns Hopkins University School of Medicine, Baltimore, Maryland, USA; 2 Division of Allergy, Immunology and Rheumatology, Department of Pediatrics, Johns Hopkins University School of Medicine, Baltimore, Maryland, USA; 3 Department of Otolaryngology-Head and Neck Surgery, Johns Hopkins University School of Medicine, Baltimore, Maryland, USA; 4 Ambry Genetics, Aliso Viejo, California, USA; 5 Department of Pediatrics, Vanderbilt University Medical Center, Nashville, Tennessee, USA

**Keywords:** autoimmune disease, Barakat syndrome, GATA3, HDR syndrome, hearing loss, juvenile idiopathic arthritis, psoriasis

## Abstract

Hypoparathyroidism, sensorineural deafness, and renal dysplasia (HDR) syndrome is caused by pathogenic variants in the *GATA3* gene located on chromosome 10p14. Here we present a 10-year-old girl with HDR syndrome who also has oligoarticular juvenile idiopathic arthritis (JIA). The patient presented for genetics evaluation with bilateral sensorineural hearing loss, oligoarticular JIA, and a renal cyst. Trio-based exome sequencing and chromosome microarray analysis revealed a pathogenic heterozygous ~1.45 Mb deletion that included the entire *GATA3* gene, consistent with a diagnosis of HDR syndrome. *GATA3* is a risk locus associated with autoimmune disease, including rheumatoid arthritis; however, the link between protein-coding variants in *GATA3* and autoimmune disease has not been well established. Previously, a single patient with HDR syndrome and psoriatic JIA was identified as having a frameshift variant in the *GATA3* gene. These reports highlight the potential for increased susceptibility to early-onset autoimmune arthritis in HDR syndrome, expanding its phenotypic spectrum. Further studies are needed to investigate the role of pathogenic variants in the *GATA3* gene in the pathogenesis of JIA and other autoimmune diseases.

## 1 | Introduction

Hypoparathyroidism, sensorineural deafness, and renal dysplasia (HDR) syndrome (MIM# 146255), also known as Barakat syndrome, is a rare autosomal dominant condition caused by haploinsufficiency of the zinc-finger transcription factor *GATA3* gene (MIM# 131320) (Upadhyay et al. 2013; Van Esch et al. 2000). The *GATA3* gene is a master regulator of T helper 2 (Th2) cell differentiation (Tindemans et al. 2014). Patients with HDR syndrome may exhibit the complete phenotypic triad of HDR syndrome or only a subset of features, the most prevalent of which is sensorineural deafness (Ali et al. 2007). Approximately 200 patients with HDR syndrome have been described in the literature (Barakat et al. 2018).

Juvenile idiopathic arthritis (JIA) is one of the most common autoimmune diseases diagnosed in children, and T cells play a central role in JIA pathogenesis (Sandborg et al. 2025). Clinical subtypes of JIA are defined by the number of involved joints in the first 6 months following symptom onset, the presence of extra-articular features, laboratory markers, and family history. JIA subtypes include oligoarticular (with one to four joints involved), polyarticular (with five or more joints involved) with the presence or absence of rheumatoid factor, psoriatic (with the presence of psoriasis in the patient or first-degree family members), enthesitis-related (with the presence of inflammation at the entheses, where bones attach to tendons and ligaments), systemic (with the presence of substantially elevated levels of inflammatory markers, fevers, and rashes), and undifferentiated (does not completely meet criteria for any one specific subtype or meets criteria for multiple subtypes simultaneously). Uveitis is the most common non-joint manifestation of JIA (Sandborg et al. 2025). Previously, psoriatic JIA was reported in a child with HDR syndrome and a pathogenic variant in the *GATA3* gene (Patrick et al. 2019).

We present a 10-year-old girl with bilateral sensorineural hearing loss, a renal cyst, and oligoarticular JIA without uveitis who was found to have a pathogenic deletion of *GATA3*, consistent with a diagnosis of HDR syndrome. Along with the previous report, this suggests an increased susceptibility to early-onset autoimmune arthritis in patients with HDR syndrome, expanding its phenotypic spectrum.

## 2 | Case Presentation

Our patient was born at 39 weeks gestation by spontaneous vaginal delivery following an uncomplicated pregnancy. Her parents were healthy, non-consanguineous, and of European descent. The patient failed her initial newborn hearing screen bilaterally but passed follow-up testing at 2 weeks of age (details of this testing are unavailable). Early developmental history was unremarkable. At 3 years, parental concern for hearing loss led to an evaluation that identified intermittent hearing loss, thought to be due to middle ear disease. At 5 years, she was diagnosed with conductive hearing loss from otitis media with effusion, leading to tympanostomy tube placement. Postoperative audiology assessment found persistent bilateral mixed hearing loss, despite functional tympanostomy tubes. Hearing loss was characterized as bilateral sensorineural hearing loss by additional testing, and she was fitted with binaural hearing aids. Follow-up evaluations demonstrated continued excellent benefit from the hearing aids, stable hearing loss, and age-appropriate speech and language skills.

At 10 years, she developed five acute episodes of right hip pain with no known injury. Each episode lasted 24–48 h and occurred in the context of upper respiratory infection symptoms without fever. On pediatric rheumatology assessment, physical examination identified mild right calf muscle atrophy and leg length discrepancy with no pain or limitation to hip range of motion. Plain films identified no osseous abnormalities.

**FIGURE 1 |:**
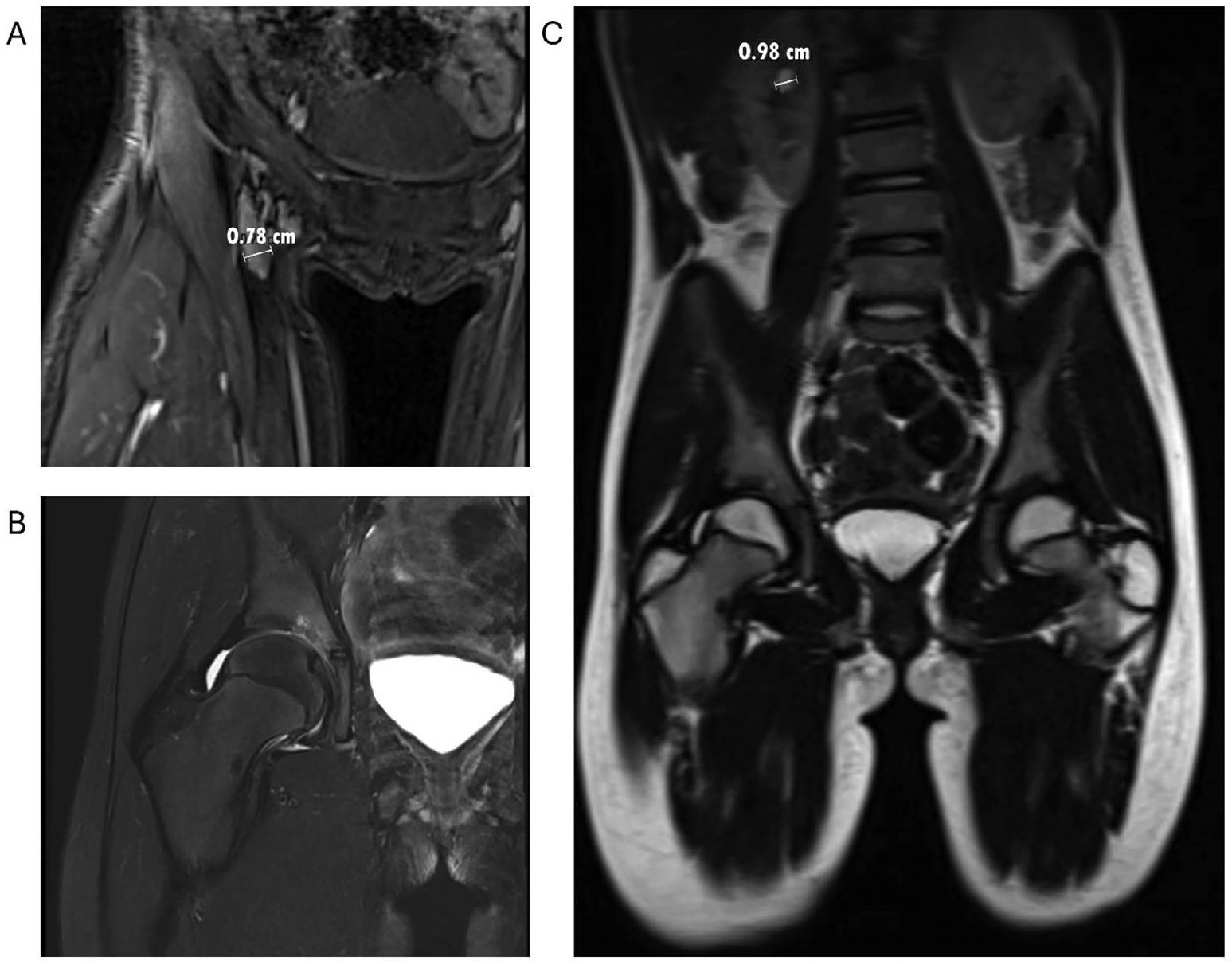
Multiplanar multisequence MRI of the right hip with and without contrast. (A, B) Small to moderate right hip joint effusion with synovitis/debris and minimal synovial enhancement. (C) Scout imaging with partially visualized right upper pole parapelvic cyst measuring approximately 1 cm.

Magnetic resonance imaging (MRI) of the right hip demonstrated an effusion with synovitis (Figure 1A,B) and incidentally found a 1 cm right upper pole parapelvic cyst (Figure 1C) that was confirmed by renal ultrasound. In these studies, there was no evidence of vesicoureteral reflux. Laboratory studies in the setting of the concurrent upper respiratory infection were significant for a weakly positive antinuclear antibody (ANA) test with a titer of 1:40, an elevated erythrocyte sedimentation rate (ESR) of 41 mm/h, and lymphopenia with absolute lymphocytes of 0.93 × 10^3^/μL. Testing for HLA-B27, rheumatoid factor, and Lyme disease was negative. She was diagnosed with ANA-positive, oligoarticular JIA. Ophthalmologic evaluation showed no evidence of uveitis.

Symptoms improved with scheduled high-dose naproxen and physical therapy. Laboratory trends showed transient increases in ESR, CRP, and urine protein and decreases in WBCs, with no significant variations. After 6 months, a repeat MRI showed persistent active right hip arthritis. Subcutaneous methotrexate, an immunosuppressive disease-modifying antirheumatic drug, was initiated, and she was referred for Genetics evaluation.

Genetics evaluation revealed no significant additional phenotyping based on history and physical examination. A three-generation pedigree was negative for a family history of hearing loss, arthritis, renal disease, congenital anomalies, intellectual disability, multiple miscarriages, and known genetic diseases. The family consented to trio-based exome sequencing and chromosomal microarray analysis.

## 3 | Genetic Analysis

Clinical trio-based exome sequencing was performed using genomic DNA from the proband and parents. Exonic regions and flanking splice junctions of the genome were captured using the IDT xGen Exome Research Panel v1.0. Massively parallel (NextGen) sequencing was completed with paired-end reads. Greater than 98% of bases had a depth of coverage of at least 20-fold, with reads aligned to the human genome build GRCh37/UCSC hg19 and analyzed for sequence variants using a custom-developed analysis tool. Additional sequencing technology and variant interpretation protocol have been previously described (Retterer et al. 2016).

Exome sequencing identified a heterozygous, *de novo* pathogenic deletion of *GATA3* (5′UTR_3′UTR) that is expected to result in the loss of function of the *GATA3* gene. To assess the deletion size, single-nucleotide polymorphism-based chromosomal microarray analysis was performed and showed two copy number losses: a pathogenic 1.455–1.466 Mb deletion at arr[GRCh37] 10p14(7,859,107_9,314,364)×1 that encompassed *GATA3* and a 130 kb deletion at arr[GRCh37] 12p12.2(20,941, 120_21,071,405)×1 of uncertain significance. The copy loss at 10p14 involved 7 genes: *TAF3, GATA3-AS1, GATA3, LINC00708, LOC105376398, LOC105755953, LOC101928272*, with only the deletion of *GATA3* known to cause a monogenic, autosomal dominant disorder. Large-scale deletions encompassing GATA3 have not been observed in population-based cohorts in the Genome Aggregation Database (gnomAD), although a patient with HDR syndrome with a *GATA3* gene deletion located on chromosome 10p15 was recently reported (Satariano et al. 2025). Loss at 12p12.2 includes 2 genes: *SLC01B3* and *SLC01B3-SLC01B7*. Alterations in *SLC01B3* are associated with Rotor type hyperbilirubinemia, a condition due to autosomal recessive digenic inheritance with the *SLC01B1* gene. A second variant in either *SLC01B3* or *SLC01B1* was not identified in exome sequencing, and the patient’s phenotype was not consistent with this condition. Thus, the 12p12.2 deletion is unlikely to be of clinical significance. Following diagnosis of HDR syndrome, the patient was referred to Endocrinology to monitor her for hypoparathyroidism and to Nephrology to monitor her for kidney disease.

## 4 | Discussion

We report a patient with HDR syndrome due to a pathogenic whole gene deletion of the *GATA3* gene with oligoarticular JIA. Two previously reported children with loss-of-function *GATA3* variants also had autoimmune diseases. One child with psoriatic JIA, bilateral sensorineural hearing loss, bilateral choanal atresia, endocrinopathies, and renal cysts was identified with a *de novo* heterozygous 2-base pair deletion in *GATA3* that caused a frameshift with stop-loss with a dominant negative mechanism (Patrick et al. 2019). Another patient with a loss-of-function *GATA3* variant developed Type I diabetes (Muroya et al. 2010). Both JIA and Type 1 diabetes are autoimmune diseases with strong T cell involvement. *GATA3* is important for increasing Th2 cells and controlling Th1 and Th17 cells (Wan 2014). Experimental studies on peripheral mononuclear cells from the child with psoriatic JIA found T cells with increased inflammatory Th1 and Th17 differentiation and decreased Th2 differentiation (Patrick et al. 2019). Taken together, these reports suggest that *GATA3* dysfunction may have broader implications for immune regulation, possibly increasing the risk of T cell-mediated autoimmune diseases.

*GATA3* is a key regulator in embryonic development and is involved in vertebrate developmental pathways (Bacha et al. 2025). In the immune system, *GATA3* is important for early thymocyte and mature peripheral T cell development (Ting et al. 1996). T helper (Th) cells include Th1, Th2, and Th17 cells, which are fundamental for clearing intracellular, helminth, extracellular, and fungal pathogens (Zhu et al. 2010). In T helper cell differentiation, *GATA3* is the master regulator for Th2 cells and suppresses the development of inflammatory Th1 and Th17 cells (Kanhere et al. 2012; Lazarevic et al. 2011; Wan 2014; Zhu et al. 2010). An increase in Th2 cell activity is associated with atopic disease, and an increase in Th1 and Th17 cell activity is associated with autoimmune and inflammatory conditions. Multiple autoimmune conditions exhibit an increase in Th1 and Th17 cell activity (Raphael et al. 2015). Th1 and Th17 cells have been identified in inflamed synovium in children and adults with autoimmune arthritis (Cope et al. 2007; Mahendra et al. 2009; Wedderburn et al. 2000). Th2 cells have been shown to have a role in suppressing inflammatory arthritis in murine models (Chen et al. 2016). Notably, *GATA3* is a susceptibility locus associated with atopic (asthma, nasal polyps, atopic derm) and autoimmune (rheumatoid arthritis, type 1 diabetes mellitus, and multiple sclerosis) diseases (Aletaha and Smolen 2018; Barnes 2008). The argument for the role of GATA3 in immune dysregulation is further supported by patients with HDR syndrome reported as affected by allergic rhinitis and chronic intestinal disorders (Lawrence et al. 2016; Rive Le Gouard et al. 2024). Yet, the mechanisms by which GATA3 contributes to immune dysregulation in this variety of diseases are not well understood, and modifying factors, including environmental and genetic risk factors, likely contribute to an individual patient’s phenotype.

Additionally, non-coding RNAs are recognized as key regulators of gene expression. Specifically, GATA3-antisense RNA 1 (GATA3-AS1) has been shown to interact with regulatory proteins and influence the expression of GATA3 (Gibbons et al. 2018). GATA3-AS1 is part of a regulatory network that influences autoimmune disease activity (Auer et al. 2022). Furthermore, GATA3-AS1 is highly expressed in clonally expanded Th2 cells in patients with type 1 diabetes (Narsale et al. 2023). Therefore, deletion of GATA3-AS1 may contribute to the observed phenotype in our patient.

The pattern of joint involvement and extra-articular manifestations can impact long-term outcomes in JIA (Shoop-Worrall et al. 2019). Predictors of disease progression include involvement of the upper limb, hip, and ankle (Nalbanti et al. 2018). JIA-associated uveitis is a common and potentially vision-threatening complication with the highest risk of occurrence in patients who are ANA-positive, have a young age of onset, and those with oligoarticular disease (Angeles-Han et al. 2013). Both reported patients with JIA and HDR syndrome had hip involvement, which suggests the possibility of a more severe disease course that might require intensive monitoring and therapy. A better understanding of the involvement of the *GATA3* gene in JIA might be helpful in identifying a more personalized therapeutic approach.

Our patient initially failed newborn hearing screening, but subsequent testing in the newborn period was reported to be normal. Concerns were later raised for conductive hearing loss, leading to placement of tympanostomy tubes; however, sensorineural hearing loss was not identified until she was 5 years of age. While hearing loss is identified in the newborn period in some individuals with HDR syndrome, others are diagnosed later in life (Lemos and Thakker 2020). Although initial reports suggested that hearing loss in HDR syndrome might be progressive (van Looij et al. 2006), a more recent cohort and systematic review suggested that the degree of hearing impairment appeared to be stable across follow-up periods (Rive Le Gouard et al. 2024).

Future directions may include longitudinal cohorts to define the prevalence of autoimmune and inflammatory features in patients with HDR syndrome and to further understand the underlying immunophenotyping. In the future, identification of pathogenic variants in the *GATA3* gene may be helpful in guiding therapies in patients with autoimmune disease. This case brings recognition to an expanding *GATA3* phenotype that could have implications for patients with HDR syndrome.

## Data Availability

The data that support the findings of this study are available on request from the corresponding author. The data are not publicly available due to privacy or ethical restrictions.
